# Supplementary figures and images for: Genome-Wide Identification and Expression Analysis of the SUT Family from Three Species of Sapindaceae Revealed Their Role in the Accumulation of Sugars in Fruits
Source: Plants (Basel). 2023 Dec 28;13(1):95. doi: 10.3390/plants13010095 (PMC10780545; doi:10.3390/plants13010095)

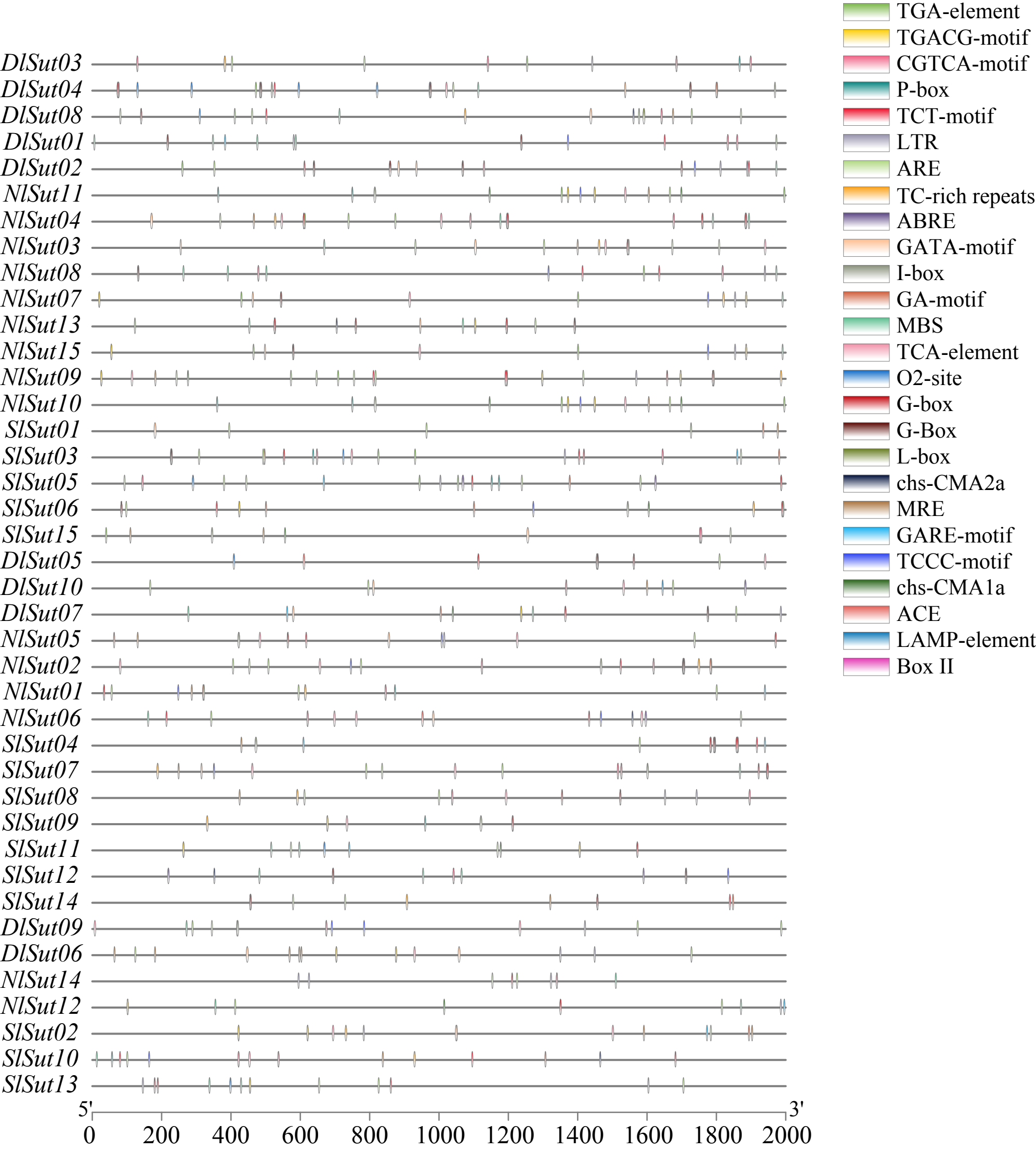

Supplement: Supplementary file 1 [file plants-13-00095-s001.zip › figure_s1.pdf]

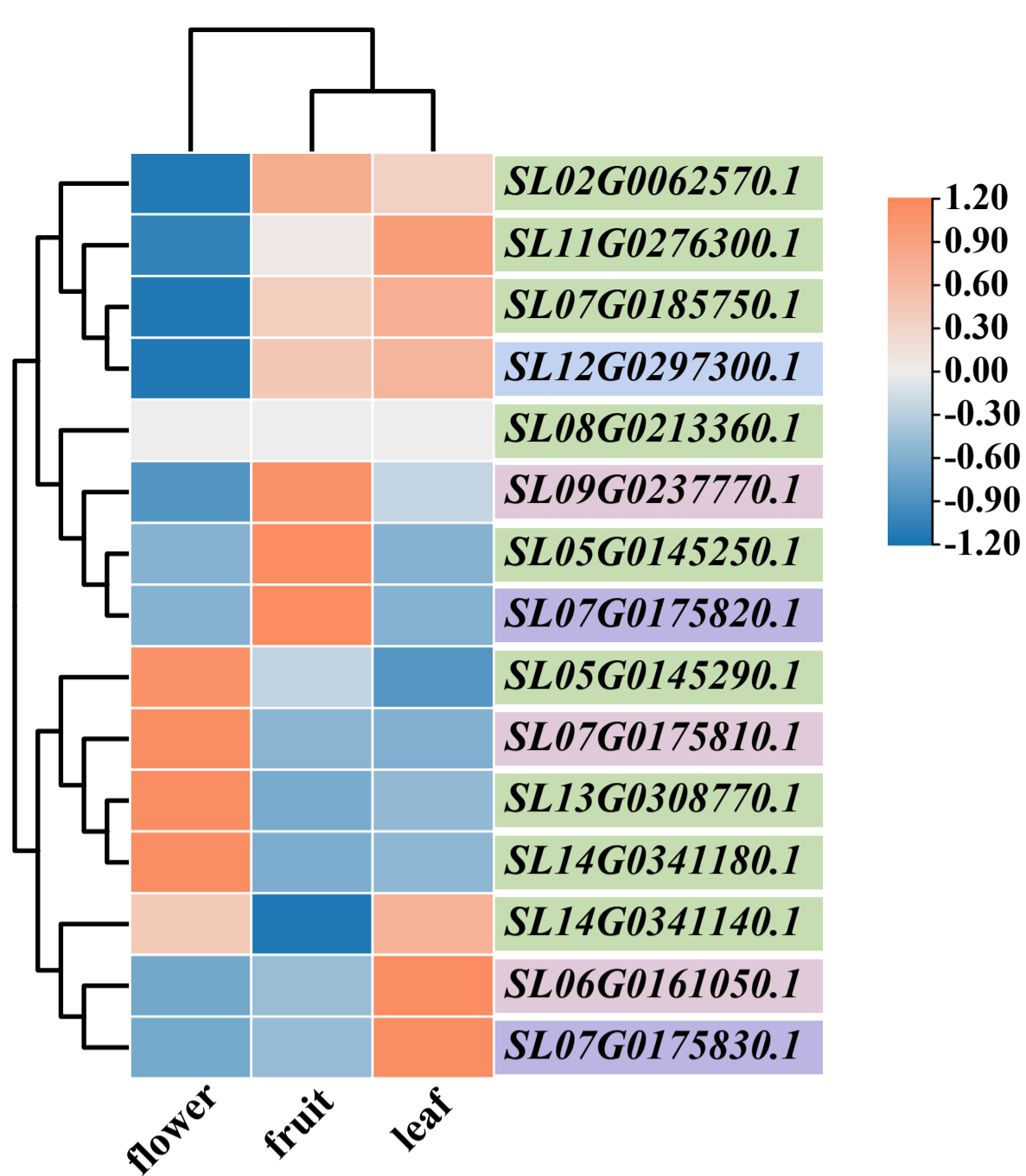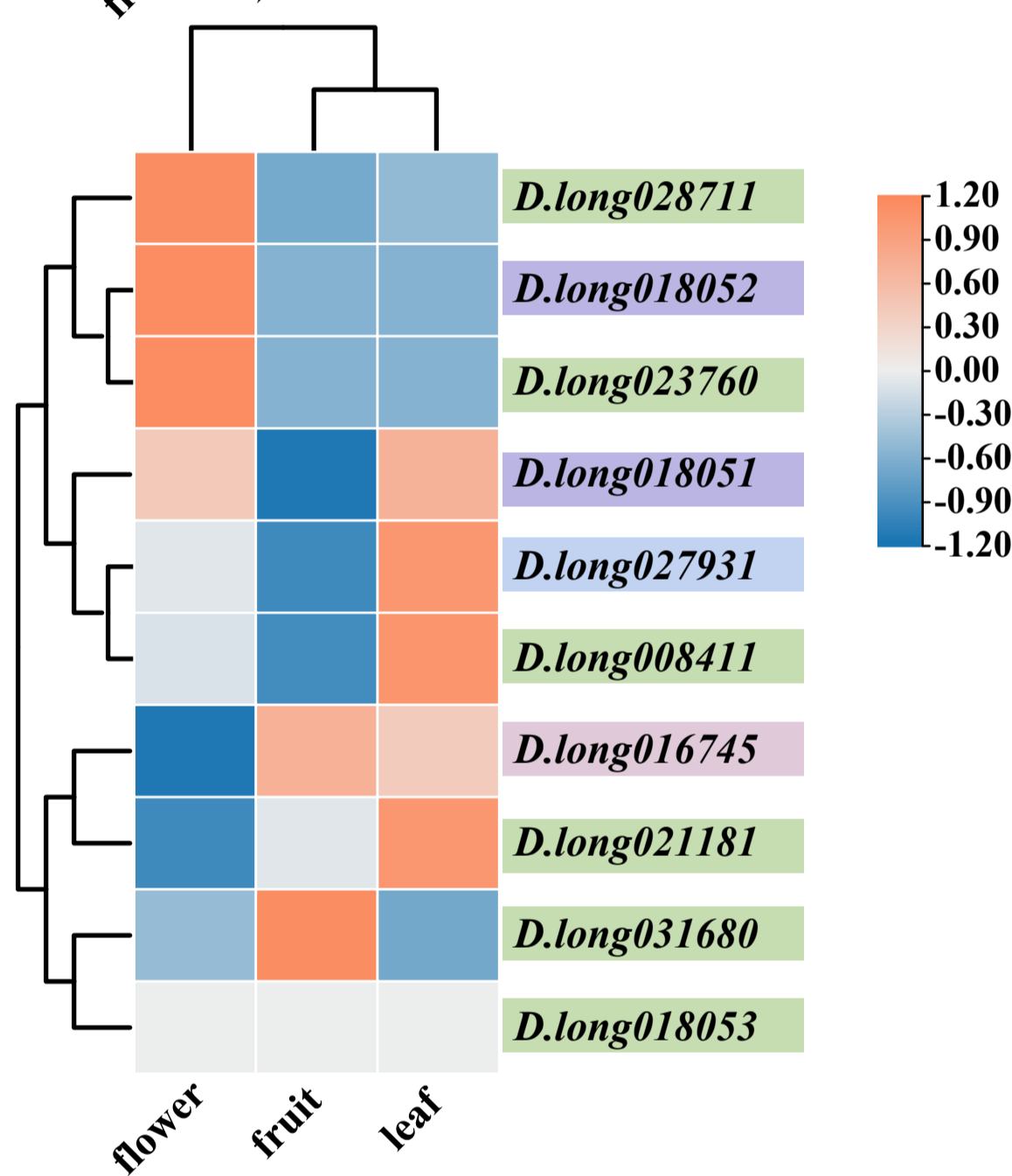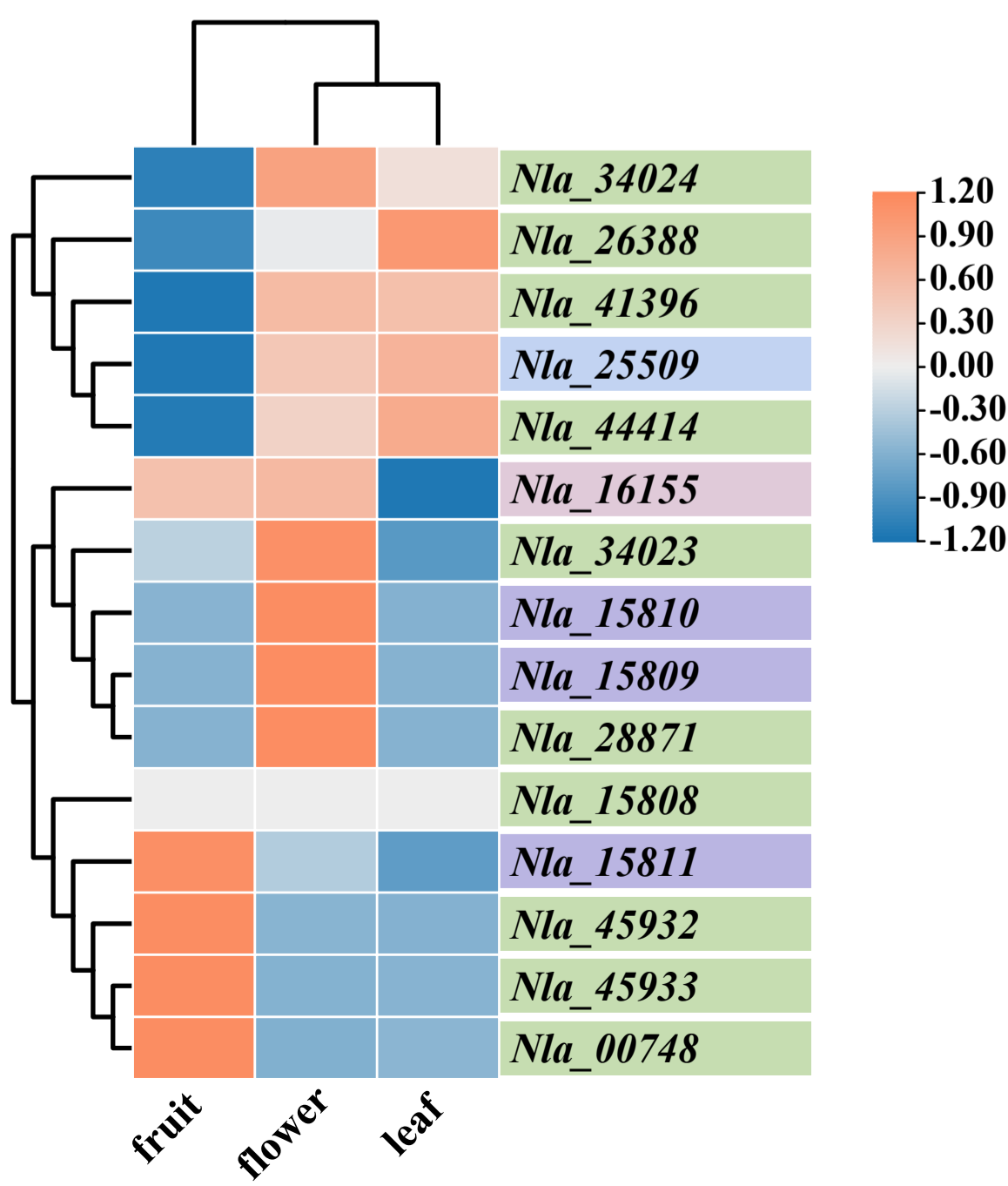

Supplement: Supplementary file 1 [file plants-13-00095-s001.zip › figure_s2.pdf]

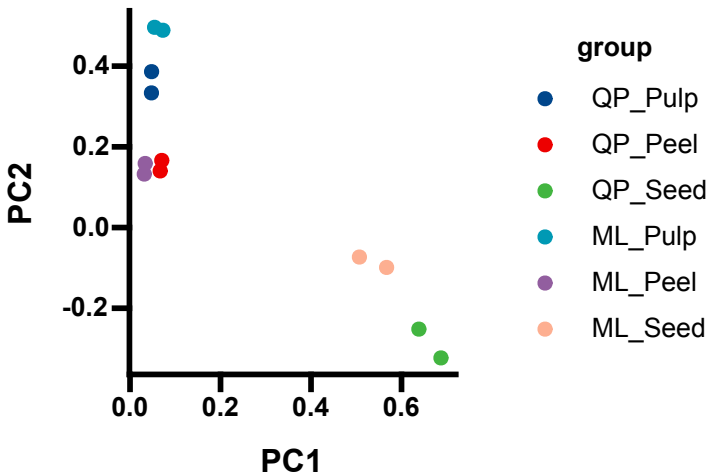

Supplement: Supplementary file 1 [file plants-13-00095-s001.zip › figure_s3.pdf]
